# Supplementary material for: The influence of the U.S. export controls against China on the resilience of Chinese corporates
Source: PLoS One. 2025 Sep 26;20(9):e0331222. doi: 10.1371/journal.pone.0331222 (PMC12469107; doi:10.1371/journal.pone.0331222)
Supplement: S1 Fig — (PDF) [file pone.0331222.s001.pdf]

Supporting Information

S1 Fig. The number of Chinese enterprises subject to export control.

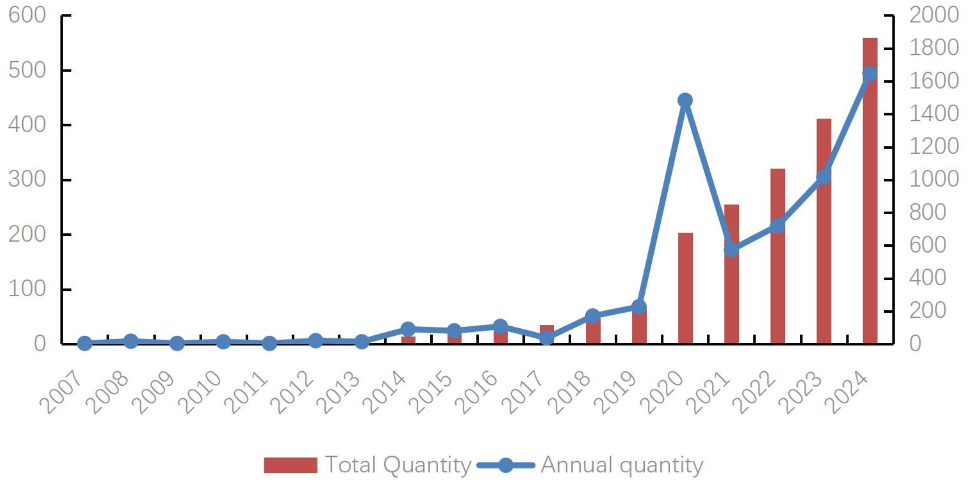

Note: The author's compilation is based on the U.S. Export Control Reform Act and the Flush iFind database.
