# Supplementary material for: The influence of the U.S. export controls against China on the resilience of Chinese corporates
Source: PLoS One. 2025 Sep 26;20(9):e0331222. doi: 10.1371/journal.pone.0331222 (PMC12469107; doi:10.1371/journal.pone.0331222)
Supplement: S2 Table — (PDF) [file pone.0331222.s002.pdf]

Supporting Information

S2 Table. Time placebo test

	(1)<br>Two years ahead	(2)<br>Three years ahead
t2	-0.0012	
	(0.0012)	
t3		0.0014
		(0.0015)
Constant	0.5821***	0.5823***
	(0.0285)	(0.0286)
Firm FE	Yes	Yes
Year FE	Yes	Yes
Observations	16133	16133
Adjusted R-squared	0.8848	0.8848

Note: Robust standard errors clustered to the firm level are reported in parentheses. *, ** and *** mean that they are statistically significant at 10%, 5% and 1% statistical level.
