# Supplementary material for: The influence of the U.S. export controls against China on the resilience of Chinese corporates
Source: PLoS One. 2025 Sep 26;20(9):e0331222. doi: 10.1371/journal.pone.0331222 (PMC12469107; doi:10.1371/journal.pone.0331222)
Supplement: S3 File — (PDF) [file pone.0331222.s003.pdf]

Supporting Information

S3 Specific Case of U.S. Tightens Tech Restrictions: FiberHome Placed on BIS Entity List

From 2018 to May 2024, the Bureau of Industry and Security (BIS) of the U.S. Department of Commerce issued a total of 37 lists of China-related entities, which cover a total of 623 Chinese companies. Fiberhome Communications (stock code SH600498), a leader in China's communications industry, has become an important target of U.S. export controls. Fiberhome is a global leader in the field of fiber optic cable and occupies a pivotal position in the global fiber optic cable industry. The importance of fiber optic cable as the cornerstone of modern communication is self-evident. Fiberhome's breakthroughs and innovations in this field have undoubtedly broken the international monopoly and made great contributions to the development of China's and even the world's communications industry. Through the representative case of Fiberhome, we will gain insights into the impact of the uncertainty brought by export control on corporate resilience, company investor sentiment, stock price volatility, capital structure and operation under the great power game.

1. Announcement Effects

Along with Fiberhome Communications in the "entity list" of enterprises, there are HUAFU Fashion Co., Ltd, NetPosa Technologies, Ltd, and 360 Security Technology Inc. 360 Security Technology Inc.'s main business is Internet advertising and services, and its revenue in 2019 amounted to 9.725 billion yuan, accounting for 76%. Fiberhome Communications is mainly

communication system equipment, with revenues amounting to 15.533 billion yuan, or 64%. Almost all of HUAFU Fashion's businesses are cotton-spinning businesses. From the performance after May 23, 360 Security Technology, HUAFU Fashion, and NetPosa shares rose slightly as a whole. From the other company's share price performance, export control for the enterprise's share price of the degree of impact is limited. Inclusion on the Entity List may also have a positive notification effect on the secondary market. Investors believed that only companies with sufficient strength would be feared by the United States and be placed on the Entity List. As a result, the three stocks as a whole rose slightly.

At the same time, from May to December 2020, the major rating agencies held Fiberhome shares with a more optimistic attitude, recommending that the stock holdings of securities companies accounted for 46%, which also let the Fiberhome investor's nervousness have been eased.

In response to the company being included in the "entity list" that may bring the impact of raw materials and core technology, Fiberhome Communications quickly issued a notice to respond. It said that its main procurement of raw materials, including general-purpose servers, boards, etc., has formulated a procurement plan and, according to the development of events, to develop a specific stocking cycle. In addition, the boards mainly come from domestic enterprises, so there is room for substitution.

After taking these forward-looking measures, Fiberhome's share price in 2022 showed a clear recovery trend, which fully demonstrated the market's recognition of the company's ability to cope with the challenges and also highlighted the company's confidence in sound operation and continuous innovation.

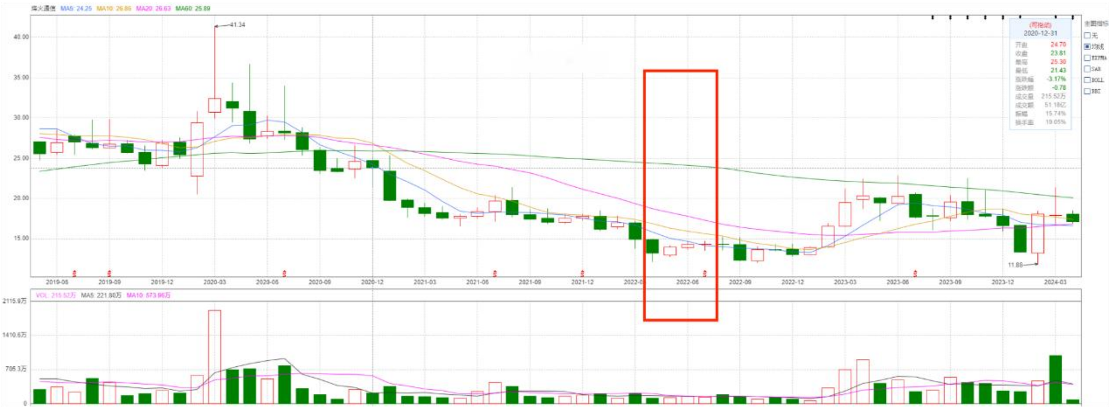

Fig 1. Fiberhome Share Price Trend

Note: Data from Oriental Wealth.

2. Strategic reserve

After being subjected to export control, Fiberhome faced a serious obstacle in the supply of key raw materials such as high-end chips. In order to cope with this challenge, Fiberhome acted swiftly and made detailed procurement plans for the main purchased raw materials, such as general-purpose servers and boards, etc., and implemented a strategic reserve strategy. At the same time, the company flexibly adjusted the specific stockpiling cycle according to the development of external events, aiming to minimize the constraints imposed by the United States on its raw material supply. As a result, starting in 2020, Fiberhome significantly increased its inventory reserves to ensure the continuous and stable operation of its business.

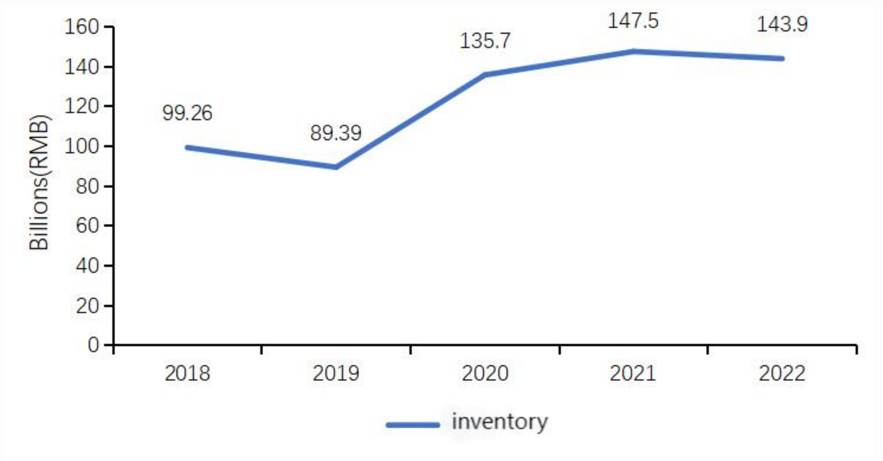

Fig 2. Fiberhome's inventory trend from 2018 to 2022

Note: Data from Oriental Wealth.

Facing the double blow of export control and the new crown epidemic, raw material prices

generally increased from 2020 onwards. However, Fiberhome has shown strong determination and strategic vision. Not only did it not retreat due to the pressure, but it also increased the stockpiling of raw materials. In addition to optical components, Fiberhome achieved a significant increase in the procurement quantity of key raw materials such as PCB circuit boards, power modules, general-purpose electronic devices and ICs. Through timely stockpiling of raw materials, Fiberhome has built a solid buffer barrier for itself to cope with emergencies, thus ensuring the stable operation of the company's production and operation for a longer period. This kind of calm and unhurried response has won more initiative and competitive advantages for it in the complex and changing market environment.

Table 1. Procurement of major raw materials for Fiberhome Communications

**Main raw materials**		2018	2019	2020	2021	2022	2023.01-03
PCB Circuit Board	Unit price (yuan/only)	127.94	123.07	148.49	41.34	32.72	28.05
	Quantity (million pieces)	147.72	174.26	394.64	695.66	745.6	184.56
	Amount (billion yuan)	1.89	2.14	5.86	2.88	2.44	0.52
Power Modules and General Electronics	Unit price (yuan/only)	2.38	2.11	1.11	0.54	0.58	0.63
	Quantity (million pieces)	7383.8	11807.08	28614.06	37426.85	5005.28	11807.99
	Amount (billion yuan)	1.76	2.49	3.19	2.01	2.9	0.75
Optical Devices	Unit price (yuan/only)	177.56	158.41	152.38	69.57	127.17	89.66
	Quantity (million pieces)	931.38	1289.7	965.98	1675.47	1052.26	300.95
	Amount (billion yuan)	16.54	20.43	14.72	11.66	13.38	2.7
IC	Unit price (yuan/only)	20.12	20.69	19.07	22.53	20.82	20.66
	Quantity (million pieces)	15824.1	16202.81	19151.97	19293.57	15099.89	3499.17
	Amount (billion yuan)	31.84	33.52	36.52	43.47	31.44	7.23

Note: Data from the official website of Fiberhome Communications.

3. Independent innovation

In 2020 and 2021, the company resolutely decided to significantly increase the R&D expenses, aiming to realize technological independence. Looking back at the past few years, from 2015 to 2018, Fiberhome Communications has maintained a steady investment in research and development, and the proportion of research and development expenditures to operating income has stabilized at about 10%. However, in the face of the sanctions dilemma in 2020, the company did not choose to retreat; instead, it chose to rise to the challenge by significantly increasing its R&D investment in order to promote the research and development of key components such as chips. This initiative led to a rapid climb in the R&D expense ratio to 14.82% and 14.16% in 2020 and 2021, demonstrating Fiberhome's firm determination and strong strength to take technological innovation as its core competitiveness in response to external pressures.

Fiberhome has always been committed to technological innovation and the enhancement of its R&D strength, and the number and percentage of its R&D personnel have shown steady growth. Especially after encountering the severe challenge of export control in 2020, Fiberhome Communications has shown strong determination and action and significantly increased the number of R&D personnel. According to statistics, the number of the company's R&D personnel increased from 7,054 to 7,526, while the percentage of R&D personnel also increased significantly to 45.29%. This initiative not only shows Fiberhome's emphasis on R&D but is also a strong reflection of the company's continuous strengthening of its independent innovation capability in order to cope with the adverse impact of U.S. sanctions.

After 2020, Fiberhome Communications Group companies have been fruitful on the road of innovation. The number of invention patents granted to the company in aggregate and the listed company itself showed a significant and encouraging upward trend. By the end of 2021, Fiberhome Communications even launched China's first 400G coherent commercial silicon optical transceiver chip by its outstanding independent R&D strength, which is a notable sign that R&D investment has been transformed into practical results. This milestone breakthrough not only highlights the company's leading position in the field of optical communications but also lays a

solid foundation for the company's future development. Along with the advancement of localization of key components, the pressure on the company's supply chain is expected to be further alleviated. By increasing investment in R&D, the company can enhance its ability to independently develop core technologies and reduce its reliance on external technologies, thereby maintaining its business operations and technological development in the face of technological blockades or export restrictions.

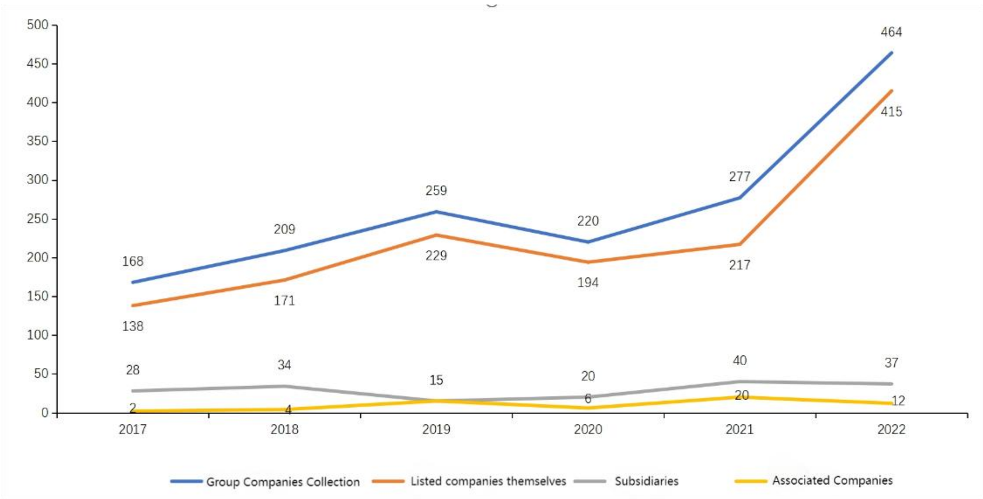

Fig 3. Number of patents for inventions independently granted during the year

Note: Data from Beacon Communications' annual report.

In the face of the challenge of sanctions, Fiberhome has demonstrated excellent expense ratio control, although R&D investment has risen in response to the demand for sanctions. Since its revenue exceeded the 5 billion mark in 2010, the company's selling, administrative and financial expense ratios have remained at relatively stable levels, roughly at 8%, 12% and 1%, respectively. However, in the first three quarters of 2023, these three expense ratios showed a more optimized trend, decreasing to 6.89%, 1.09% and 0.96%, respectively.

Fiberhome has always regarded R&D as the core driver of the company's development. Since 2009, the Company's R&D expense ratio has been consistently maintained at the level of 10%, reflecting the company's long-term commitment and strong belief in technological innovation. Especially after the sanctions in 2020, the company did not retreat due to difficulties; instead, it invested more resources in R&D to break through the key technological bottlenecks by cutting

down sales expenses. This initiative not only highlights the company's wise decision on cost control but also highlights the company's great importance and firm determination on independent R&D and innovation. By increasing investment in R&D, Fiberhome is continuously promoting technological progress and laying a solid foundation for the company's long-term development.

4. Expanding Overseas Markets

According to the 2019 annual report, Fiberhome's revenue in 2019 was 24.662 billion yuan, of which 6.77 billion yuan, accounting for 27.45%, so after being included in the "Entity List", Fiberhome's overseas industry will be hit to some extent. From the perspective of Fiberhome's global service network, the company's global service network is mainly located in Southeast Asia, along the Belt and Road, Africa, South America and other regions. Europe and the United States have no service network. Therefore, the U.S. sanctions will have an impact on the company's overseas business expansion, but the degree of impact is limited. Being included in the "entity list" forced Fiberhome Communications to vigorously increase the number of research and development personnel and research and development expenditure. In addition, Fiberhome Communication has also increased its efforts to expand overseas sales markets, enrich international trade channels, and promote the optimization of enterprise supply chain structure.

Fiberhome Communications will expand its sales network around the world, with the domestic market as the core, and in more than 50 countries around the world, to establish a comprehensive sales and service system. Its products and services cover more than 100 countries and regions, of which the Southeast Asia and South American markets have become the focus of its overseas business. By 2022, the revenue of the three major sectors will reach 20.92 billion yuan, 6.509 billion yuan and 3.31 billion yuan respectively, accounting for 67.65%, 21.05% and 10.13% of the total revenue. Especially with the active development of the Southeast Asian and South American markets, the proportion of revenue from overseas markets has been further improved.

In the face of the challenge of U.S. sanctions, Fiberhome demonstrated resilience in the overseas market. After being subjected to sanctions in 2020, the company's overseas revenue from its main business not only did not decline but instead realized significant growth. In 2021, after the sanctions, Fiberhome's overseas revenue amounted to 9.212 billion yuan, with a year-on-year

growth rate of 34%, a growth rate that significantly exceeded the level of overseas revenue growth during 2018-2020. The company's overseas revenue share also continued to rise, reaching 35.01% in 2021, an increase of 7.56 percentage points compared to pre-sanctions 2019.

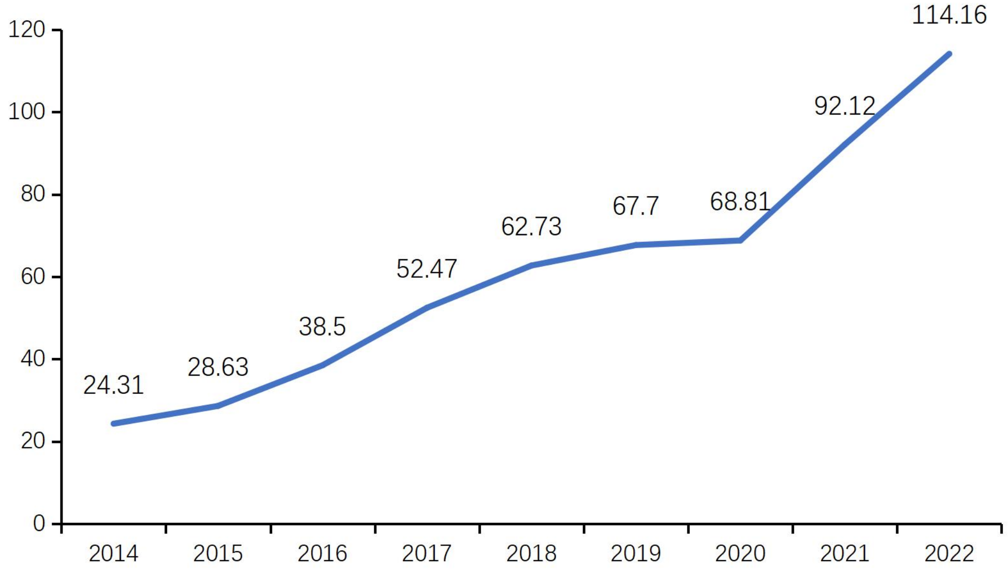

Fig4. Fiberhome's Overseas Main Business Revenue, 2014 to 2022

Note: Data from Cathay Pacific database.

Successful expansion in overseas markets brought Fiberhome significant growth in total operating income. In 2021, the company's operating revenue reached 26.315 billion yuan, up 25% year-on-year, successfully returning to the growth track. Entering 2022, Fiberhome continued to maintain strong momentum from Q1 to Q3, realizing operating revenue of 21.528 billion yuan, up 16% year-on-year. After being included in the "Entity List" in 2020, Fiberhome's operating income from its three major segments of communication systems, fiber optic cables and cables, and data network products declined for a while. However, the company quickly took a series of anti-sanctions measures to effectively respond to the external challenges so that the revenue of these three segments gradually rebounded. By 2022, the revenue of the three segments reached $20.92 billion, $6.509 billion and $3.131 billion respectively, accounting for 67.65%, 21.05% and 10.13% of the total revenue in that order. From the perspective of revenue structure, Fiberhome demonstrated a relatively stable trend, proving that export control did not significantly affect the company's revenue structure.

Conclusion

How should enterprises deal with export control? Fiberhome puts forward coping strategies for raw material reserve, R&D innovation, investor sentiment appeasement, and overseas market development. First, in terms of raw material reserves, Fiberhome Communications has increased the strategic reserves of raw materials to enhance the resilience of the supply chain. By increasing raw material inventory, the company can better absorb the impact of supply chain disruption and reduce the dependence on a single supplier, thus reducing the risk of production delays or stagnation caused by supply problems. Second, in terms of R&D and innovation, Fiberhome has substantially increased R&D expenses and cultivated R&D talents. The company's patents have been greatly enhanced, China's first 400G coherent commercial silicon optical transceiver chip has been launched, and the R&D investment has begun to bear fruit. Third, in terms of investor appeasement, in response to investor concerns, a positive response to appease investor sentiment is firm investor confidence, so the stock price has recovered. Fourth, Fiberhome mitigates the risk of sanctions and reduces its dependence on a particular market by increasing its overseas share and adopting diversified markets.
