# Supplementary material for: The influence of the U.S. export controls against China on the resilience of Chinese corporates
Source: PLoS One. 2025 Sep 26;20(9):e0331222. doi: 10.1371/journal.pone.0331222 (PMC12469107; doi:10.1371/journal.pone.0331222)
Supplement: Attachment — Submitted filename: Response to viewers.docx [file pone.0331222.s004.docx]

**Response to viewers**

Dear Editor and Reviewers,

Thank you very much for your comments regarding our manuscript entitled "*The* *Influence of the U.S. Export Controls Against China on the Resilience of Chinese Corporates*". We are truly grateful to your critical comments and thoughtful suggestions on how to improve our manuscript. The valuable comments and criticisms have provided important guidance for our research and the revision of our manuscript. Based on these comments and suggestions, we have made appropriate modifications on the original manuscript, then we hope to meet your approval. The revisions themselves are addressed point-by-point in the subsequent text.

# Response to Reviewer #1

1). The placement of the organization of the study between the introduction and the theoretical framework disrupts the flow of information. I suggest to remove organization of the study to ensure a smoother transition and clearer progression of ideas.

**Response:** We sincerely thank the reviewers for their valuable comments on the flow of information in the research organisation section of the paper. We fully agree with the suggestion to remove the research organisation section and have removed it at the end of the introduction in the first part of the revised paper. As shown in the manuscript (P6).

2). To improve the logical flow of the introduction, start by providing an overview of the research topic, followed by defining key concepts. Then, link the study to relevant theories, highlighting the theoretical gap in existing literature. Next, identify the empirical gap, explaining the limitations of previous research in addressing your specific focus. Finally, clearly state the objective of the study, outlining its contribution to filling these gaps. This structure ensures a smooth progression from context to the research's purpose and significance.

**Response:** Thank you very much for your professional comments on our article. According to your suggestions, we have optimized the structure of the introduction section and specifically modified it as follows (P2-P6) :

①To address the conceptualization issue, after outlining the topic and background of the study, we added conceptual definitions of export control and business resilience. In particular, the definition of export control adopts the relevant conceptual definitions in the U.S. Export Administration Regulations (EAR). Described as "*Export controls, as delineated by the Export Administration Regulations (EAR)*……" (P3).

The definition of firm resilience is based on Sajko's (2020) statement. Described as "*Existing research has defined business resilience as an important ability of a business entity to cope with external shocks and secure long-term growth (Sajko, 2020).* " (P5) .

②Regarding the shortcomings of existing literature, after elaborating the theoretical gaps in existing literature, we added the current limitations of empirical research. Described as "*From the viewpoint of the current state of empirical research, although there has been a gradual increase in research on trade policy and enterprise resilience in recent years.* …… " (P5) .

③With regard to the significance and contribution of the research, after explaining the limitations of previous studies on export control and firm flexibility, we sort out the purpose, contribution and significance of the research in the last paragraph of the introduction. Paper's contribution to filling existing research gaps is outlined in the innovation points and contributions. Described as "*Compared to prior studies, this paper's potential innovations and contributions mostly lie in the following areas.* ……"	(P6).

3). After presenting the theoretical framework, it is crucial to include an empirical review that synthesizes key studies examining the effects of trade policies, particularly export controls, on corporate resilience and internationalization.

**Response:** We sincerely thank the reviewers for their valuable suggestions. We have expanded the Literature Review section by adding a literature review of relevant empirical studies (P5), which summarizes the key studies examining the impact of trade policy (especially export control) on firm resilience and internationalization. In addition, in the Theoretical Mechanisms Analysis and Research Hypotheses section, we add relevant studies on trade policy uncertainty, export control, and firm resilience (P7-P8) and add more references (references [23]-[35]) to support the article's ideas. Specific modifications are as follows:

1. The literature review of the complementary empirical studies is presented as " *From the viewpoint of the current state of empirical research, although there has been a gradual increase in research on trade policy and enterprise resilience in recent years.* ……" (P5).
2. The supplementary related literature research is as follows " *Despite the gradual increase in research on trade policy and enterprise resilience in recent years* ……" (P7-P8):
3. Additional references are given. Described in the part of "*References* " (references [23]-[35]):

4). It is recommended to include an extensive description of the data used in the study, detailing the sources, scope, and characteristics of the sample, while also discussing the potential benefits, such as the comprehensive coverage of A-share manufacturing companies, and limitations, such as potential biases or data gaps, to provide a clearer understanding of the data’s relevance and reliability.

**Response:** We thank the reviewers for your careful review of the data description of this article. In response to your comment ‘need to add data sources, sample characterisation and discussion of strengths and weaknesses’, we have systematically improved the data section based on the original article. Described in the part of *" 3.3 Data sources "* (P18-P20).

1. Address the issue of sample source. The US export control data in this paper comes from the official website of the US BIS and is manually sorted out, the original data of the enterprise's explanatory variables and control variables comes from the CSMAR database, and the mechanism variables and moderating variables come from the CSMAR, WIDN and other authoritative databases and are further calculated by the authors, and the data source of each variable in this paper is labelled in the table below. CSMAR and WIND databases are recognised as the most authoritative and comprehensive data sources in the field of China's capital market research, and their data collection process strictly follows the public information such as annual reports of listed companies and stock exchange announcements, and after multiple cross-checks, their reliability has been widely recognised by academics at home and abroad.
2. Data scope and characteristics. This paper mainly focuses on the analysis of China's manufacturing enterprises, according to the ‘Guidelines for Industry Classification of Listed Companies (Revised in 2012)’ issued by the China Securities Regulatory Commission, the manufacturing industry category includes 31 categories from C13 to C43, including, but not limited to, machinery manufacturing, electronic equipment manufacturing, chemical industry, pharmaceutical manufacturing, etc., which is able to reflect the overall situation of China's manufacturing industry in a more comprehensive manner. It can reflect the overall situation of China's manufacturing industry in a more comprehensive way. According to the classification of listed companies in the CSMAR database, the sample of enterprises in 2015-2023 covers 29 major categories of enterprises in the manufacturing industry, while C16 (tobacco products industry) and C43 (metal products, machinery and equipment repair industry) are naturally excluded due to the absence of listed companies. The enterprise sample in this paper also covers data from 29 broad categories of enterprises in the manufacturing industry, essentially achieving comprehensive coverage of manufacturing enterprises.
3. To address the issue of potential advantages of the data, in the industry dimension, the sample of this paper focuses on the manufacturing industry, which is an important pillar of the national economy and occupies a key position in the national economy. At the same time, this paper takes Shanghai and Shenzhen A-share listed manufacturing companies as the research sample, which can reflect the situation of representative enterprises in China's manufacturing industry in a more comprehensive way; in the time dimension, the sample of this paper spans 2015 - 2023, during which China's manufacturing industry has experienced rapid development and transformation and upgrading, and at the same time, faces external challenges such as U.S. export control. The longer time span helps us observe the changes in firms' resilience in different economic environments and policy contexts, and capture the dynamic adjustment process of firms before and after the implementation of the export control policy; in terms of data processing, this paper takes a series of rigorous steps, such as excluding firms with financial anomalies, firms with consecutive observations less than three years old, and the samples with missing main variables, and conducting Winsor shrinkage on the continuous variable processing. These processing measures help to control the effects of outliers and data noise and ensure the quality of the data and the stability of the model estimation.
4. To address the issue of potential limitations of the data, despite our rigorous screening and processing of the samples, there may still be some bias. For example, this paper mainly selects A-share listed companies as samples, and there may be sample bias because these companies are relatively larger and have better governance structures, which may not fully represent all Chinese manufacturing enterprises, especially a large number of small, medium and micro enterprises. During data processing, we excluded samples with missing main variables, and missing data may lead to selective bias in the sample, affecting the generality and accuracy of the research results. In addition, due to the timeliness and availability of the data, we may not be able to fully capture all the factors that may affect firm resilience, which may lead to some bias.

5.) The findings would benefit from a discussion of potential causality concerns and omitted variable bias.

**Response:** We sincerely thank the reviewers for their valuable comments.

In response to the potential causality issue you mentioned, we would like to explain how the design of this study avoids the potential causality issue and add relevant discussions to enhance the rigour of the argument. The core explanatory variable in this study is U.S. export control policy (with whether an industry is included in the Entity List as a proxy variable), while the explanatory variable is the resilience performance of Chinese firms. The update of the Entity List is led by the Bureau of Industry and Security (BIS) of the U.S. Department of Commerce. In terms of the logic of policy formulation, decisions on U.S. export control policies are mainly based on macro-strategic considerations such as U.S. national security and technological competition, rather than the business conditions or resilience levels of individual Chinese firms. Meanwhile, this paper adopts the implementation policy as a quasi-natural experiment. Policy implementation comes before, and changes in enterprise resilience come after. Chinese firms are unable to influence the U.S. government's regulatory decisions by adjusting their own resilience levels in a reverse direction, which mechanistically excludes the possibility of reverse causation. Described in the part of "3.2.2 Explanatory variables" (P15).

In order to further discuss the issue of potential causality rigorously in terms of empirical research, we have taken three approaches to conduct robustness tests in the article. The first method is the PSM-DID, as described in the revised version "*4.3.1 PSM-DID test"* (P23-P24). The second method is the placebo test, which takes both the individual firm placebo test and the time placebo test, see the revised draft "*4.3.2 Placebo test*" (P24-P26) for details. The third method is to lag the control variables by one period for regression analyses, as detailed in the revised draft "*4.3.3 Other robustness tests"* (P26-P27). The results of the studies all prove that the impact of US export control policy on Chinese firm resilience is exogenous. See the revised draft (P23-28) for details:

The issue of omitted variables has been discussed in ‘4.3.3’. ‘In order to avoid estimation bias caused by omitted variables, this paper includes firms' invention patents and government subsidy variables in the regression equation.’ And it is found that the findings of this paper are still robust after considering the omitted variables, as detailed in "*4.3.3 Other robustness tests* " (P26-P28) of the revised version.

6.) It is recommended to provide a clear justification for the selection of control variables by explaining how each variable is relevant to the research question and how it helps isolate the effect of US export controls, rather than merely listing them, while also discussing why other potential factors were excluded, based on their irrelevance to the study’s focus or potential for introducing confounding effects.

**Response:** Thank you to the reviewers for their valuable suggestions. We fully agree with you about the control variables in the article. We have added the relevant content in the third point of the first part of the description of the measures and data in Chapter 3, Research Design, in the revised version of the paper. Described in the part of "*3.2.3 Control variables*" (P15-P18).

7.) It is recommended to clearly state the source of each table within the paper, specifying whether the data is derived from primary research, publicly available datasets.

**Response:** We thank the reviewers for their valuable suggestions. We have labelled the data sources below all tables (Tables 1-9) in the revised version of the paper (where the data in Tables 2 and 3 are exactly the same as those in Table 1, and we will not repeat the description) (P19-P41).

8. ）It is recommended to explicitly state the study's limitations, particularly regarding any data constraints, such as sample size, data availability, or potential biases, and address possible measurement errors that could affect the validity of the results, ensuring a transparent and balanced interpretation of the findings.

**Response:** Thank you to the reviewers for their valuable suggestions. We fully agree with you on the control variables in the article. We have added a fourth section on study limitations for clarification in Chapter 3, Study Design, in the revised version of the paper. Described in the part of "6. *Conclusions and policy recommendations*" (P42-43), as "*We list a few limitations to this study……*".

9.) It is recommended to include suggestions for further development of the field by exploring additional dimensions, such as the long-term impact of export controls on firm innovation, comparing the resilience of firms across different countries, and incorporating qualitative case studies to better understand strategic responses to trade disruptions, all of which would provide richer insights and help refine future research in the area.

**Response:** Thank you for the reviewer's valuable suggestions. In response to your suggestion of "deepening the research through more dimensions", we have carried out in-depth discussions and made additional improvements, and now we would like to explain the relevant modifications and reflections as follows:

1. Provide recommendations for further development of the field by exploring additional dimensions. Following the reviewers' comments, this paper explores the impact of more dimensional factors on firm resilience. Considering the supply chain as the core link of firm's operations, the effectiveness of its structure and management directly determines the firms’ ability to adapt and recover in the face of external shocks, this paper adds a comparative study of the resilience performance of firms with different supply chain concentration in coping with export control. Described in the part of "*5.2.5 Heterogeneity analysis based on supply chain concentration*" (P39-P40).
2. Analysis of the long-term impact of export controls on enterprise innovation. In response to this recommendation, this paper makes the following attempts to replace corporate toughness with corporate innovation indicators (corporate R&D investment, patent applications, patent grants, etc.), and the regression results find that the impact of export controls on corporate innovation is not significant, and it also fails to pass the parallel trend test, which may stem from two levels of reasons: first, due to the dynamic adjustment characteristics of the U.S. export control lists, enterprises tend to offset control impacts through short-term adaptive strategies such as supply chain One, due to the dynamic adjustment characteristics of the U.S. export control list, enterprises tend to offset the control shocks through short-term adaptive strategies such as supply chain reconstruction and technology substitution, so the impact on corporate innovation is not significant; two, innovation activities have strong path dependence, the cycle of major technological breakthroughs is usually longer, and the technological blocking effect of export controls may take longer to be transformed into innovation incentives, the U.S. has strengthened its export controls on China since 2018, which has lasted only about five years so far, and the long-term effect of corporate innovation may take longer to be visible. effects may take longer to emerge. In the future, we will continue to promote the study of the long-term impact of export controls on corporate innovation by expanding the time span of data.
3. Comparison of the resilience of firms in different countries. This study focuses on the specific policy context of "U.S. Export Controls on China" and the impact of U.S.-China technological decoupling on Chinese firms, so the data and scope of the study mainly focus on U.S.-China export controls and Chinese manufacturing firms. Cross-country comparisons would require revising the title of the paper and collecting relevant data from firms in other countries, taking into account factors such as the economic environment, policy context, and firm structure of different countries, which is beyond the scope of this paper. At the same time, we believe that the direction of expansion pointed out by the reviewers is very inspiring for our further in-depth research, and that this comparison is important for understanding the coping strategies of global firms in the face of similar trade disruptions. In the future, we will continue to promote research in this area by establishing a multinational research team and collecting data from global firms.
4. Incorporation of qualitative case studies. We sincerely thank you for your valuable comments and constructive suggestions on this paper. In response to your suggestion to supplement the qualitative case study, we have added the analysis of the qualitative case study in the discussion of theoretical mechanisms section (P8-P10) to enhance the empirical support of the theoretical framework. Specifically, the case reveals the relevant impact of Fiberhome Communications after the export control, as well as the strategic response to the trade disruption. To facilitate readers' access to the details, the complete description of the case's data sources and analytical process has been organized in Supporting information. The appendix's content closely echoes the logic of the main text. To avoid the length of the main text, the relevant details are presented here as supplementary materials.

# Response to Reviewer #2

The study "The Influence of the U.S. Export Controls Against China on the Resilience of Chinese Corporates" is topical and engaging, presenting the context of escalating U.S.-China trade frictions and how the new U.S. export control measures are affecting Chinese enterprises. Through the methodology employed, including the use of a multi-period difference-in-differences model and the analysis of data from listed companies in China's manufacturing sector, the paper highlighted the negative impact of controls on corporate resilience, identifying conditions under which companies can mitigate these effects.

My specific observations and recommendations are:

1. In the Introduction section:

a) while mentioning the impact of export controls on corporate resilience, it should be clearly emphasized how this research differs from previous studies. What are the new or unique aspects of this approach in analyzing the impact of export controls.

**Response:** Thank you very much for your valuable comments and suggestions on the thesis. We have carefully read your review comments and have revised and improved the paper according to your suggestions. In response to your suggestions in the introduction section, we have supplemented the literature review section with an analysis of the limitations of existing studies in terms of the impact of export controls on firm resilience (P5) and the last paragraph of the introduction with a compendium of the paper's innovations and research contributions (P6). These modifications aim to highlight more clearly the differences between this study and previous studies, as well as the unique perspective of this study in analyzing the impact of export controls.

b) simplify some sentences that are too long to improve readability and clarity of the text.

**Response:** We sincerely appreciate your valuable suggestions. We have asked a professional agency to re-touch the article and simplify the overly long sentences. The revised manuscript has better readability and clarity.

c) introduce some details on how and why these export controls have evolved over time, linking them to specific policy and technological changes, to provide a deeper understanding of the context.

**Response:** Thank you very much for your valuable suggestions on the paper. In response to your comment on "Supplementing the contextual analysis of export controls over time", we have supplemented and improved the research context in the introduction as follows (P2):

1. Analysis of Policy Evolution: We have added a discussion of the Export Control Reform Act (ECRA), elaborating on the idea that it signalled a strategic shift in U.S. export control policy from a traditional security orientation to one driven by technological competition. This addition is intended to link the dynamic evolution of export controls to the changing landscape of global technological competition, providing a deeper policy context to support the study. Specific additions are as "*With the changes in the geopolitical landscape and the intensification of technological competition* ……"(P2).
2. Technology-driven logic: By introducing the dynamic perspective of Sino-U.S. technological competition, we analyze the reality that the scope of U.S. export controls on China significantly expands with the escalation of technological competition. Specific additions are as "*With China's development in AI and semiconductor manufacturing, the U.S. has included AI chips and advanced process equipment* ……"(P2).
3. Data visualization: To strengthen the argument, we included in the Supporting information a graph of the change in the number of Chinese enterprises subject to US export controls between 2007 and 2024 (S1 Fig). Further support for the core point that "the number of Chinese enterprises sanctioned by the US export control list is increasing".

*S1 Fig. The number of Chinese enterprises subject to export control.*

|  | Total Quantity | Annual quantity |
| --- | --- | --- |
| 2007 | 1 | 1 |
| 2008 | 6 | 5 |
| 2009 | 7 | 1 |
| 2010 | 11 | 4 |
| 2011 | 12 | 1 |
| 2012 | 18 | 6 |
| 2013 | 22 | 4 |
| 2014 | 49 | 27 |
| 2015 | 73 | 24 |
| 2016 | 105 | 32 |
| 2017 | 116 | 11 |
| 2018 | 167 | 51 |
| 2019 | 235 | 68 |
| 2020 | 680 | 445 |
| 2021 | 852 | 172 |
| 2022 | 1068 | 216 |
| 2023 | 1373 | 305 |
| 2024 | 1867 | 494 |

*Note: The author's compilation is based on the U.S. Export Control Reform Act and the Flush iFind database.*

2. In the Analysis of theoretical mechanisms and research hypotheses section :

a) in section 2.1, explain why US export controls affect production efficiency and technological costs for Chinese companies, providing specific examples or case studies;

**Response:** Thank you for reviewing our paper in your busy schedule. In response to your valuable suggestions, we have added the case of Fiberhome Communication Technology Co. in Section 2.1 (P8-P10) to illustrate how U.S. export control affects the productivity and technology cost of Chinese firms with specific examples. Specific case details have been presented as supplementary materials in Supporting information.

b) diversify the source of studies cited to support the claims and increase the robustness of the arguments;

**Response:** Thank you very much for your valuable comments and suggestions on the paper. In order to enhance the persuasiveness of our arguments, we have supplemented 21 authoritative literatures in the analysis of theoretical mechanisms and research hypotheses section (P6-P13), which enriches the theoretical framework from different perspectives and further supports our analysis. These literatures cover the impact of export control on enterprise internationalization, enterprise financing, enterprise risk, etc., which provide theoretical support for the mechanism of export control's impact on enterprise resilience.

c) introduce a discussion of long-term strategies that companies can adopt to address the negative effects of export controls, not just short-term responses or temporary adaptations.

**Response:** Thank you for reviewing the paper and for your suggestions. In response to your suggestion to supplement the qualitative case study, we have added the analysis of the qualitative case study in the "*2.1 Export controls and business resilience*" (P8-P10) to enhance the empirical support of the theoretical framework. Specifically, the case reveals the relevant impact of Fiberhome Communications after the export control, as well as the strategic response to the trade disruption. To facilitate readers' access to the details, the complete description of the case's data sources and analytical process has been organized in Supporting information. The appendix's content closely echoes the logic of the main text. To avoid the length of the main text, the relevant details are presented here as supplementary materials.

3. In Section 3:

a) why sales revenue growth and the standard deviation of monthly stock returns are most relevant for measuring corporate resilience.

**Response:** We thank the reviewers for their careful review of the indicator variables in this paper. We believe that the original meaning of resilience is to recover and bounce back, while firm resilience refers to the ability of firms to resist shocks, to be able to recover and bounce back, and to resume development in the face of shocks from unfavourable events. As such, we combine corporate market performance and operating conditions to measure a firm's shock resistance and long-term resilience. Specifically, we use the ‘standard deviation of monthly stock returns’ to reflect this reflective resilience. Stock price volatility directly reflects the market's evaluation of firms' risk exposure. In the case of regulatory shocks, lower volatility indicates greater investor confidence in the stability of the firm. We use ‘sales revenue growth’ to reflect the long-term resilience of firms. The fact that a firm can still achieve revenue growth after a regulatory shock indicates that it has recovered its business vitality through market substitution, product adjustment and other strategies. Described in the part of " *3.2.1 Explained variable* " (P14).

b) explain how each control variable was chosen.

**Response:** Thank you to the reviewers for their valuable suggestions. We fully agree with your views on the control variables in the article. We have added an explanation of how each control variable was selected in the third point of the first part of the description of the measures and data in Chapter 3. Described in the part of "*3.2.3 Control variables*" (P15-P18).

c) in describing the data selection process, what is the reason for excluding ST, *ST and PT firms to clarify how this affects the integrity of the sample.

**Response:** We thank the reviewers for their careful review of the data selection process of this paper. Regarding the reasons why ST, *ST and PT category companies are excluded and their impact on sample completeness, we have added further explanations to the original article as follows and described in the part of "*3.3 Data sources*" (P18-P20):

① rationale for excluding ST, ST and PT type companies

In China's A-share market, companies in the ST (Special Treatment), *ST (Delisting Risk Warning) and PT (Particular Transfer, delisted but providing special transfer services) categories have the following characteristics:

First, financial anomalies or operational risks: such companies are usually specially labelled by the Exchange for continuous losses, major violations or insolvency, and their financial data may not reflect normal operating conditions.

Second, delisting or restructuring uncertainty: *ST and PT-type companies are on the verge of delisting or have already been delisted and may be undergoing unconventional operations such as asset restructuring and government bailouts, and their strategies for coping with export controls may be systematically different from those of healthy companies.

Third, market trading constraints: the share prices of such companies are highly volatile and illiquid, and related indicators (e.g., market capitalisation, yield) may be distorted, interfering with quantitative analyses of corporate resilience.

For the above reasons, the exclusion of these firms in this paper is to:

On the one hand, to improve sample homogeneity: to ensure that the study focuses on normal operating firms and to avoid the interference of outliers on statistical results (e.g., regression analysis);

On the other hand, to enhance the robustness of the conclusions: the extreme risk of ST/*ST/PT companies may mask the general mechanism of export control impact on corporate resilience, and the exclusion better reflects the coping ability of "typical companies ".

1. Impact of the exclusion of ST, ST and PT companies on the sample completeness

According to CSRC data, during the period 2015-2023, the sample of ST, ST and PT firms among all A-share listed companies accounts for about 2.8%, and the sample of ST, ST and PT firms among all A-share listed companies in the manufacturing sector accounts for about 4.6%. The sample size accounts for a relatively small proportion of the sample, and the exclusion still covers the vast majority of normal operating companies, is representative at the industry and scale levels, and will not affect the overall distribution of the sample as well as the core conclusions.

4. In Section 4:

a) Although the explanatory variables are said to be significantly positive, in Table 2, the DID coefficients are negative. This seems to contradict the statement that the impact is positive. This discrepancy needs to be clarified to properly explain the meaning of the impact of the variables on firms' resilience;

**Response:** We thank the reviewers for their careful review of the full text presentation. In Table 2, the double-difference coefficient is negative, but the explanatory variables are said to be significantly positive in the textual presentation. After checking, it is an error in the author's presentation, which has been corrected in the baseline regression analysis of the empirical analysis in Part IV of the paper, as follows (P20-P21):

Described as "*The analysis reveals statistically significant negative coefficients (p<0.01) for both the key explanatory variables and export control measures, indicating that Entity List designations substantially reduce corporate resilience.* "

b) When checking for robustness using the PSM-DID test, how were the variables selected for the propensity score calculation;

**Response:** Thank you for your careful review of this paper. Regarding the issue of ‘the basis of variable selection for propensity score calculation’ raised in the robustness test, in order to maintain the comparability of the models and to ensure the consistency of the two types of models in the logic of causal identification, this paper adopts the same control variables for regression as those of the baseline DID model in the propensity score-matched double-difference (PSM-DID) test. benchmark DID model with the same control variables for regression, specifically including firm size, leverage, age at listing, return on net assets, firm growth, current ratio, cash holdings, price-earnings ratio, proportion of independent directors, proportion of chairman's shareholding, nature of equity, and Herfindahl index as the matching variables. Described in the part of "*4.3.1 PSM-DID test*" (P23-P24).

c) The interpretations and conclusions drawn from the placebo test are not clearly related to the study objectives.

**Response:** We thank the reviewers for their careful assessment of the methodological rigour of this article. Regarding your valuable comment that "the relevance of the placebo test findings to the study objectives is not clear ", we have reflected deeply and responded to your comments in detail at three levels: study design, methodological logic, and interpretation of the results, in order to further clarify the relevance of the placebo test to the study objectives. Described in the part of "*4.3.2 Placebo test*" (P24-P26).

1. Illustration of the logical relevance of the individual firm placebo test

The objective of this study is to identify the causal effect of U.S. export controls on the resilience of Chinese firms. However, possible unobserved omitted variables in the empirical analysis (e.g., other political and economic policy disturbances, etc.) can lead to biased estimation results. For this reason, we design placebo tests with randomly assigned pseudo-experimental groups, whose core logic is to verify the robustness of the benchmark regression results by constructing counterfactual scenarios. Specifically:

Test design: if the effects of export controls are real, their effects should be reflected only in the actual regulated firms (the experimental group); conversely, if other omitted variables are the main drivers of changes in firm resilience, the effects of these variables are likely to be generalisable, and will be present irrespective of whether the firms are realistically regulated or not. Therefore, we construct a pseudo-experimental group (i.e., we randomly select firms as "falsely regulated firms") through 500 random self-help samples and repeat the estimation of the "pseudo-effect" of export controls.

Interpretation of results: Figure 3 of the paper shows that the coefficient estimates for the randomly assigned experimental group are clustered around zero, and the vast majority of the estimates are insignificant at the 10 per cent confidence level (the estimates from the true benchmark regression are significantly negative and not included in the placebo distribution). This suggests that the spurious policy effect in the pseudo-experimental group should converge to zero if the export control policy itself is an exogenous shock and is not confounded by other omitted variables. Thus, the results of the placebo test support the conclusion of the benchmark regression that the reduction in firm resilience is indeed driven by export controls per se and not by other unobserved confounders.

Relevance to the study's objective: This test responds to the potential question of ‘whether the effects of export controls are confounded by other policy or economic factors’, thus strengthening the chain of causal inference between export controls and business resilience, in line with the study's objective of "identifying the direct effects of export controls". consistent with the study's objective of identifying the direct effects of export controls.

② Explanation of the logical relevance of the time-placebo test

The purpose of the time placebo test is to verify whether the baseline results may be driven by time trends or inherent differences in business resilience rather than real policy effects. The specific logic is as follows:

Test design: if the change in firm resilience stems from an exogenous shock of export controls, the policy effect should be significant only at the true policy point in time (e.g., the year the entity list was issued); if the baseline results are driven by differences in the time trends inherent in firms in the experimental and control groups (e.g., firms in the experimental group are already in the channel of declining resilience), the regression results may still be significant.

Interpretation of results: Appendix 2 shows that none of the estimated coefficients for advancing the policy timing are significant (in contrast to the significant negative effect of the benchmark regression). This suggests that changes in firm resilience cannot be predicted by the fictitious policy time point, and that significant effects emerge only at the real policy time point.

Relevance to research objectives: This test rules out the possibility that ‘the difference in firm resilience between the experimental and control groups is dominated by time trends’, thus further supporting the core finding that "export control shocks are causally related to changes in firm resilience".

.

5. The "Further analysis" section exhaustively covers the mechanisms through which export controls influence firms' resilience. Suggestions and corrections to be made to improve the clarity and accuracy of the text:

a) In the introduction of section 5.1, it is mentioned that export control "will reduce" instead of "reduces" or "has reduced", which would be more appropriate to reflect observed effects and not predictions or generalizations.

**Response:** We thank the reviewers for their valuable grammatical suggestions. We fully acknowledge your proposed changes and have modified the relevant tenses in the introduction of Section 5.1 of the revised version of the paper as follows (P28):

Described as "*The theoretical analysis in the previous section shows that the export control has reduced the level of firm resilience by inhibiting firm internationalization, shrinking the scale of financing, and exacerbating firm risk*"*.*

b) The term "toughness" used to describe firms' resilience seems unclear. Perhaps "resilience" or "capability to withstand external shocks" would be more appropriate, as they are more specific and easier to understand.

**Response:** Thank you for the reviewer's valuable suggestions on terminology vocabulary. We fully acknowledge your proposed changes and have revised the terminology in Section 5.1.1 of the revised version of the paper. Described as "*It shows that the resilience of firms with lower internationalization decreases more significantly than firms with higher internationalization, i.e., the export control reduces the resilience of firms by inhibiting firms' level of internationalization……*" (P29).

c) Table 5 describes the effects of the variables through the interaction with DID, but it would be useful to clearly explain how each of these variables is conceptualized and measured before presenting the results. For example, what do "Overfirm" and "Oversold" represent before discussing their results.

**Response：**Thank you very much for your advice on on the clarity of variable definitions in this paper. We fully agree with the fundamental role of variable conceptual clarification in the interpretation of empirical results and have made the following refinements in the paper. Described in the part of "*5.1.1 Inhibitory effects of firm internationalization*" (P28-P30).

d) When discussing political connectedness and strategic aggressiveness, provide more details on how these are assessed and why they are relevant to the study of resilience to export controls;

**Response：**Thank you for your constructive comments on the clarity of the variable descriptions in this paper as well as the logic of the paper. We have added and refined the measures of correlation between political affiliation and strategic aggressiveness and the correlation with firms' resilience in coping with export controls, which have been revised in the heterogeneity analyses further. Described in the part of "*5.2.3 Heterogeneity analysis based on political affiliation*" (P36-P38) and "*5.2.4 Heterogeneity analysis based on analyst attention*" (P38-P39).

e) In the analysis based on analyst attention and political connectedness, explain why these characteristics may influence how firms respond to export controls by also referring to recommendations from other countries and economies(https://doi.org/10.1080/21665095.2022.2032237;http://www.transformations.knf.vu.lt/53a/article/expo; https://doi.org/10.1515/ev-2023-0014; DOI: 10.15838/esc.2022 .6.84.4). How can analysts improve risk management or how political connections can facilitate access to resources in times of trade restrictions?

**Response：**Thank you very much for the strong support to our work. We fully acknowledge your questions. We have added relevant content to the section based on analyst focus and political relevance in the revised version of the paper. Described in the part of "*5.2.2 Heterogeneity analysis based on analyst attention*" (P35) and "*5.2.3 Heterogeneity analysis based on political affiliation*" (P36-P38).

We tried our best to improve the manuscript and made some changes in the revised version of the paper. We sincerely appreciate the time and effort that the reviewers have invested in evaluating our manuscript. We are eager to receive any additional feedback or suggestions that may further enhance our work.
